# Identification of Epigenetic Biomarkers of Lung Adenocarcinoma through Multi-Omics Data Analysis

**DOI:** 10.1371/journal.pone.0152918

**Published:** 2016-04-04

**Authors:** Chie Kikutake, Koji Yahara

**Affiliations:** 1 Division of Biostatistics, Kurume University School of Medicine, Fukuoka, Japan; 2 Biostatistics Center, Kurume University, Kurume, Fukuoka, Japan; 3 Institute of Life Science, College of Medicine, Swansea University, Swansea, United Kingdom; Inc, UNITED STATES

## Abstract

Epigenetic mechanisms such as DNA methylation or histone modifications are essential for the regulation of gene expression and development of tissues. Alteration of epigenetic modifications can be used as an epigenetic biomarker for diagnosis and as promising targets for epigenetic therapy. A recent study explored cancer-cell specific epigenetic biomarkers by examining different types of epigenetic modifications simultaneously. However, it was based on microarrays and reported biomarkers that were also present in normal cells at a low frequency. Here, we first analyzed multi-omics data (including ChIP-Seq data of six types of histone modifications: H3K27ac, H3K4me1, H3K9me3, H3K36me3, H3K27me3, and H3K4me3) obtained from 26 lung adenocarcinoma cell lines and a normal cell line. We identified six genes with both H3K27ac and H3K4me3 histone modifications in their promoter regions, which were not present in the normal cell line, but present in ≥85% (22 out of 26) and ≤96% (25 out of 26) of the lung adenocarcinoma cell lines. Of these genes, *NUP210* (encoding a main component of the nuclear pore complex) was the only gene in which the two modifications were not detected in another normal cell line. RNA-Seq analysis revealed that *NUP210* was aberrantly overexpressed among the 26 lung adenocarcinoma cell lines, although the frequency of *NUP210* overexpression was lower (19.3%) in 57 lung adenocarcinoma tissue samples studied and stored in another database. This study provides a basis to discover epigenetic biomarkers highly specific to a certain cancer, based on multi-omics data at the cell population level.

## Introduction

The epigenetic mechanism is vital in regulating gene expression. Failure of the epigenetic modifications can result in inappropriate activation or inhibition of various signaling pathways and lead to diseases such as cancer [1–3]. Cancer cells experience dramatic epigenetic changes, including CpG dinucleotide hypermethylation and loss of acetylation, leading to tumor suppressor genes (TSG) downregulation and, on the other hand, pronounced hypomethylation of the promoter regions of oncogenes and microsatellite regions that lead to their activation [4].

Epigenetic alterations, including aberrant DNA methylation in the promoter and alterations in histone modifications, can be used as epigenetic biomarkers of cancer diagnosis and promising targets for epigenetic cancer therapy [5] that aims to reverse cancer-specific epigenetic alterations to a more normal epigenetic state [6]. Several epigenetic drugs such as decitabine (DNA hypomethylating agents for all subtypes of myelodysplastic syndromes) and vorinostat (histone deacetylase inhibitor for the treatment of leukemia) have already been approved by the Food and Drug Administration (FDA) and others are currently being tested in clinical trials [7].

However, epigenetic alterations have physiological functions in both normal and cancer cells [5]. Thus, there are concerns regarding the accuracy of epigenetic diagnostic or potential side effects of epigenetic therapies [8, 9]. Therefore, high specificity to cancer cell is a difficult and important problem for epigenetic therapies to achieve.

Toward the development of epigenetic therapies with higher cancer cell specificity, a recent genome-wide study [5] explored DNA methylation and H3K27me3 histone modification that are more frequent in human cancer cells than in normal cells. In the study, the frequency of methylated genes, which also presented H3K27me3 (i.e., dual modification) was higher in colon, breast, and prostate cancer cell lines than in normal cells. Another study [10] presented an approach that combined both epigenetic and genetic (sequence-specific ATFs) strategies to reactivate regions that were epigenetically downregulated in metastatic tumors.

However, the recent genome-wide study that reported the dual modifications [5], was conducted by using microarrays to analyze DNA methylation, H3K27me3 histone modification, and gene expression across a genome. In addition, the study did not focus on a specific type of cancer. In order to find epigenetic biomarkers with high specificity in a certain type of cancer, in this study, we analyze multi-omics data, including ChIP-Seq and RNA-Seq of 26 lung adenocarcinoma cell lines and a normal cell line, Small Airway Epithelial Cell (SAEC). We extended the target to six types of histone modifications (H3K27ac, H3K4me1, H3K9me3, H3K36me3, and H3K27me3 in addition to H3K4me3) across a human genome, including regions that cannot be analyzed by microarray. We also explore associations between the histone modifications and aberrant gene expression that are highly specific to lung adenocarcinomas. Moreover, we validated our findings by utilizing ChIP-Seq data of another normal lung cell line and RNA-Seq data of another set of 538 paired samples (tumor and normal, 1076 in total) from the same cancer patients.

We determined that *NUP210* gene encoding a main component of the nuclear pore complex presented both H3K27ac and H3K4me3 histone modifications in its promoter regions among ≥85% (22 out of 26) and ≤96% (25 out of 26) of the lung adenocarcinoma cell lines. *NUP210* was the only gene not showing the dual modifications in either SAEC or another normal cell line, Normal adult Human Lung Fibroblasts (NHLF). It was also one of the two genes showing aberrant overexpression among all 26 lung adenocarcinoma cell lines compared to the normal cell line SAEC, although the extent of overexpression was usually smaller in the other paired datasets. The results indicate that *NUP210* gene is the most promising epigenetic biomarker for lung adenocarcinoma.

## Materials and Methods

### Identification and Annotation of Regions with Histone Modifications

We used ChIP-seq data of 26 lung adenocarcinoma cell lines and SAEC from the DBTSS database (S1 Table) [11]. To identify the regions of histone modifications, “peaks” of ChIP-Seq tags were called by MACS2 for the six types of histone modifications (H3K27ac, H3K4me3, H3K4me1, H3K9me3, H3K36me3, and H3K27me3) in each cell line. Narrow peaks (H3K27ac, H3K4me3, and H3K4me1) were detected by MACS2 with default options. Broad peaks (H3K9me3, H3K36me3, and H3K27me3) were detected by MACS2 using “-broad” and “-nomodel” options [12] and specifying “-q (q-value)” to be 0.05 according to the official website of MACS (https://github.com/taoliu/MACS/).

For each lung adenocarcinoma cell line, we extracted histone modifications that were not detected in SAEC. From the histone modifications, we calculated frequency of shared histone modifications among the 26 lung adenocarcinoma cell lines (as a population) by bedtools version 2.22.1 [13]. These histone modifications were annotated in terms of closest genes and distances to Transcriptional Start Sites (TSS) of the genes by the package of ChIPseeker [14] in R version 3.1.0 when they were found in ≥85% (22 out of 26) among the lung adenocarcinoma cell lines. We focused on genes with the histone modification in their promoters defined as up to ±1.5 kb from the most upstream TSSs [15].

### Examination of the Histone Modifications in Another Normal Cell Line

We examined ChIP-Seq data of NHLF, another only one normal lung cell line for which ChIP-Seq experiments have been conducted and their peaks were inferred and made publicly available by Bernstein–Broad Institute (http://genome-mirror.duhs.duke.edu/ENCODE/cellTypes.html). We examined whether the histone modifications for genes of interest in lung adenocarcinoma cell lines are also absent in NHLF cells.

### Analysis of Gene Expression Levels

We examined the expression levels of genes of interest in each of the 26 lung adenocarcinoma cell lines and SAEC by using RNA-Seq data stored in the DBTSS database. We examined which gene was aberrantly expressed in which lung adenocarcinoma cell line according to the following criteria [15]: (i) genes with ≥4- or ≤1/16-fold reads per kilo base per million (RPKM) of SAEC if the genes were transcribed (>1 RPKM) in SAEC and (ii) >5 RPKM if the genes were not transcribed (≤1 RPKM) in SAEC. We counted the number of lung adenocarcinoma cell lines presenting aberrant gene expression.

### Examination of Chromatin States

We examined chromatin states of the promoters defined as up to ±1.5 kb from the most upstream TSSs [15] in the genes of interest by using information of the eight types of states. They were inferred along a genome in the previous study [15] by using ChromHMM [16] and stored in the DBTSS database: (i) active promoter; (ii) weak/poised promoter; (iii) strong enhancer; (iv) weak enhancer; (v) transcriptional elongation; (vi) inactive region; (vii) inactive region/heterochromatin, and (viii) low/no signal. These eight states were illustrated using following colors; red, pink, orange, yellow, green, blue, purple, and white, respectively. Chromatin states, which appeared most frequently in each promoter, were drawn for each lung adenocarcinoma cell line and SAEC. Two chromatin states were shown by using two colors when they appeared with similar frequency.

### Analysis of the Distribution of Single Nucleotide Polymorphisms in Promoter and Gene Bodies

We examined the distribution of Single Nucleotide Polymorphisms (SNPs) in the promoters defined as up to ±1.5 kb from the most upstream TSSs [15] and gene bodies (exons and introns) of the genes by using data of Single Nucleotide Variants (SNV) stored in the DBTSS database. For each gene of interest, we explored a SNP potentially shared among the 26 lung adenocarcinoma cell lines.

### Analysis of the Expression Levels of Histone Modifying Enzymes

We focused on the following histone lysine methyltransferases, histone lysine demethylases, histone acetyltransferases, and histone deacetylases, which are associated with the six types of histone modifications. We compared their expression levels (in RPKM) in each of the 26 lung adenocarcinoma cell lines with those of SAEC. The fold-changes of expression levels were analyzed according to the following criteria: genes with ≥2- or ≤1/4-fold RPKM of SAEC if the genes were transcribed (>0 RPKM) in SAEC.

### Validation in Terms of Gene Expression by Using Other Datasets

We conducted validation in terms of aberrant gene expression. We examined RNA-Seq data of 538 pairs of tumor compared with those of normal tissue samples from the same cancer patients, which are available in the BioXpress database (https://hive.biochemistry.gwu.edu/tools/bioxpress/) [17] collected from The Cancer Genome Atlas (TCGA: http://cancergenome.nih.gov/) for the following 11 cancers and carcinomas, including lung adenocarcinoma: lung adenocarcinoma [Lung_Ade], lung squamous cell carcinoma [Lung_Squ], rectum adenocarcinoma [Rectum_Ade], colon adenocarcinoma [Colon_Ade], prostate adenocarcinoma [Prostate_Ade], breast invasive carcinoma [Breast_Inv], thyroid carcinoma [Thyroid], kidney renal papillary cell carcinoma [Kidney_Pap], kidney renal clear cell carcinoma [Kidney_Cle], kidney chromophobe [Kidney_Chr], and liver hepatocellular carcinoma [Liver_Hep]. These consist of 57, 50, 6, 26, 50, 113, 59, 30, 72, 25, and 50 paired samples, respectively (S2 Table), all of which were obtained by the RNASeq Version 2 experiments according to TCGA. For each gene in each sample, the extent of differential expression in the cancer cells compared to the normal cells is recorded in the database. We also used baseline expression data for each gene in 7 different tissues, breast, colon, kidney, liver, lung, prostate, and thyroid (S2 Table), in the BioXpress database collected from Expression Atlas (https://www.ebi.ac.uk/gxa/baseline/experiments) and examined whether the aberrant gene expression in lung adenocarcinoma cell lines is also detected in a large dataset using the following criteria [15]: (i) genes with ≥4- or ≤1/16-fold RPKM of each normal tissue if the genes were transcribed (>1 fragments per kilo base per million (FPKM)) in normal tissues from baseline expression data, and (ii) >5 RPKM if the genes were not transcribed (≤1 FPKM) in normal tissues from baseline expression data. We also examined to what extent the aberrant gene expression was specific to lung-adenocarcinoma when compared to the remaining 10 cancers and carcinomas.

## Results

### Histone Modifications Highly Specific to Lung Adenocarcinoma

To identify genes presenting epigenetic modifications highly specific to lung adenocarcinoma, we analyzed ChIP-Seq data of six types of histone modifications (H3K27ac, H3K4me3, H3K4me1, H3K9me3, H3K36me3, and H3K27me3) in 26 lung adenocarcinoma cell lines and SAEC [15] stored in the DBTSS database [11]. The “peaks” of ChIP-Seq tags were called by MACS2 for the six types of histone modifications in each of the 26 lung adenocarcinoma cell lines. Similarly, “peaks” for the six types of histone modifications in SAEC were called and used as a control in the following analyses. The number of histone modifications detected in each lung adenocarcinoma cell line and not observed in SAEC was counted. The average number of each of the six lung adenocarcinoma-specific histone modifications among the 26 lung adenocarcinoma cell lines was 19,691 (H3K27ac), 5,149 (H3K4me3), 32,908 (H3K4me1), 18,113 (H3K9me3), 23,938 (H3K36me3), and 43,275 (H3K27me3), respectively (S1 Fig and S3 Table).

From the histone modifications detected in at least one lung adenocarcinoma cell line, but not in SAEC, we calculated the frequency of shared histone modifications among the 26 lung adenocarcinoma cell lines (as a population). We extracted those that were detected in equal to or more than 85% (22 out of 26) of the cell lines in the promoter regions defined as up to ±1.5 kb from the most upstream TSSs [15] and conducted further analyses. We identified 182 genes with H3K4me3 histone modifications and 10 genes with H3K27ac in the promoter regions (Table 1). Lists of genes, the histone modifications, and fold enrichment of the peaks are shown in S4 (for H3K4me3) and S5 Tables (for H3K27ac).

**Table 1:** Number of genes with lung adenocarcinoma-associated chromatin marks in the promoter regions.

	Frequency among the 26 lung adenocarcinoma cell lines	
chromatin marks	≥85%, but ≤96%	100%
H3K27ac	10	0
H3K4me3	178	4
H3K27ac & H3K4me3	6	0
H3K4me1	0	0
H3K9me3	0	0
H3K36me3	0	0
H3K27me3	0	0

**Fig 1:**
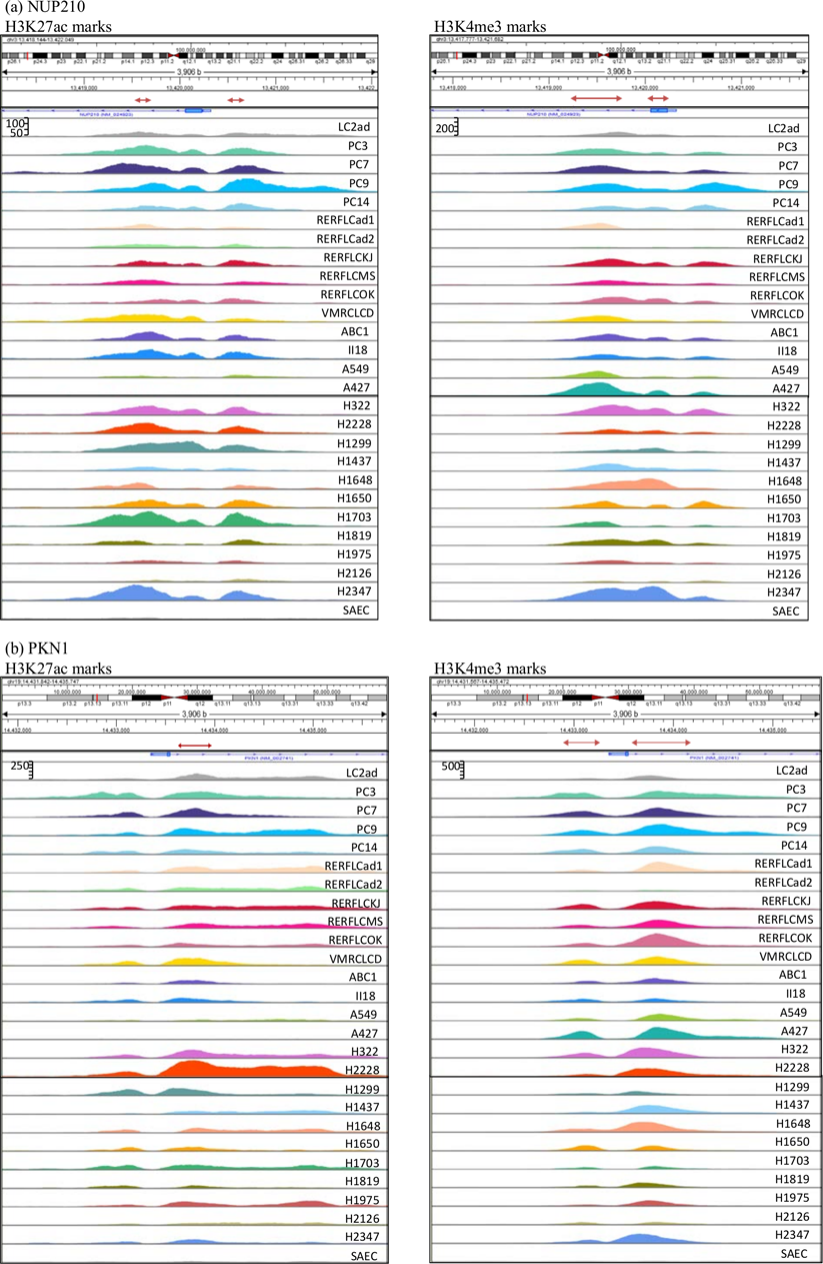
Read coverage of H3K27ac and H3K4me3 histone modifications. H3K27ac and H3K4me3 histone modifications highly specific to lung adenocarcinoma in 26 lung adenocarcinoma cell lines are located in the promoter regions of (a) *NUP210* and (b) *PKN1*. The left panel represents H3K27ac and the right panel represents H3K4me3 histone modification. The blue arrows indicate the location of the *NUP210* and *PKN1* genes and the red arrows indicate regions of histone modifications. Small Airway Epithelial Cell line (SAEC) at the bottom is a normal control cell line, while the others are 26 lung adenocarcinoma cell lines. H3K27ac and H3K4me3 read coverage is shown in a range of 0–150 and 0–400 in (a) and 0–250 and 0–500 in (b), respectively. Similar illustrations of the other four genes in Table 3 are shown in S3 Fig. The genomic positions in this figure are based on hg38.

Among them, as indicated in Table 1, four genes with H3K4me3 histone modifications shared in all 26 lung adenocarcinoma cell lines were identified (Table 2). We also identified six other genes carrying both the H3K27ac and H3K4me3 marks that are highly specific to lung adenocarcinoma (Table 3). Read coverage of the H3K27ac and H3K4me histone modifications for two out of the six genes (*NUP210* and *PKN1*) is shown in Fig 1(A) and 1(B) as an example.

**Table 2:** Four genes carrying the lung adenocarcinoma-associated H3K4me3 histone modification in the promoter regions among all cell lines.

Gene	Description	Functions	Known relationship to cancer
*NFE2L3*	nuclear factor, erythroid 2 like 3	Transcription factor, implicated in carcinogenesis, stress response, differentiation, and inflammation. (Chevillard and Blank 2011, Kannan, Dodard-Friedman et al. 2015)	Colorectal cancer [18]
*ETV4*	ets variant 4	ETS transcription factor (Oh, Shin et al. 2012)	Breast cancer, Ewing tumors, prostate cancer, colorectal cancer, gastric cancer, esophageal cancer, ovarian cancer, oral cancer, lung cancer (non-small cell lung cancer) [19]
*PRTG*	protogenin	Immunoglobulin superfamily protein (Ito, Nakamura et al. 2011, Wang, Juan et al. 2013)	Colorectal cancer [20]
*TMEM86A*	transmembrane protein 86A	unknown	-

**Table 3:** Six genes carrying lung adenocarcinoma-associated dual histone modification of H3K27ac and H3K4me3 in the promoter regions.

Gene	Description	Functions	Known relationship to cancer
*NUP210*	nucleoporin 210kDa	Main component of the nuclear pore complex, forming a gateway that regulates the flow of macromolecules between the nucleus and the cytoplasm.	See Discussion
*PKN1*	protein kinase N1	Regulation of the intermediate filaments, the actin cytoskeleton, cell migration, and tumor cell invasion.	Ovarian cancer [21], colorectal cancer [22], prostate cancer [23, 24], breast cancer [25], gastric cancer [26]
*PPP1R9A*	protein phosphatase 1, regulatory subunit 9A	Synapse formation and control of actin cytoskeleton reorganization.	Prostate cancer [27]
*IGF2BP3*	insulin-like growth factor 2 mRNA binding protein 3	Induction of cell proliferation and invasiveness via posttranscriptional regulation of IGF2.	Various cancer [28–30]
*HOXC4*	homeobox C4	Transcription factor and progression of lymphoid differentiation.	Prostate cancer [31, 32]
*FAM102B*	unknown	unknown	-

The read coverage was much higher in most of the 26 lung adenocarcinoma cell lines than that in SAEC. The read coverage of the other genes is shown in S2 (for the genes in Table 2) and S3 Figs (for the other genes in Table 3).

### Examination of the Chromatin Marks in Another Normal Cell Line

The analyses above depend on a single non-carcinoma cell line, SAEC. In order to validate that the histone modifications are highly specific to lung adenocarcinoma, we examined whether they were also absent in another normal cell line, NHLF. All four genes in Table 2 carried H3K4me3 histone modifications in their promoter regions (±1.5 kb from TSS) in NHLF (S4 Fig).

**Fig 2:**
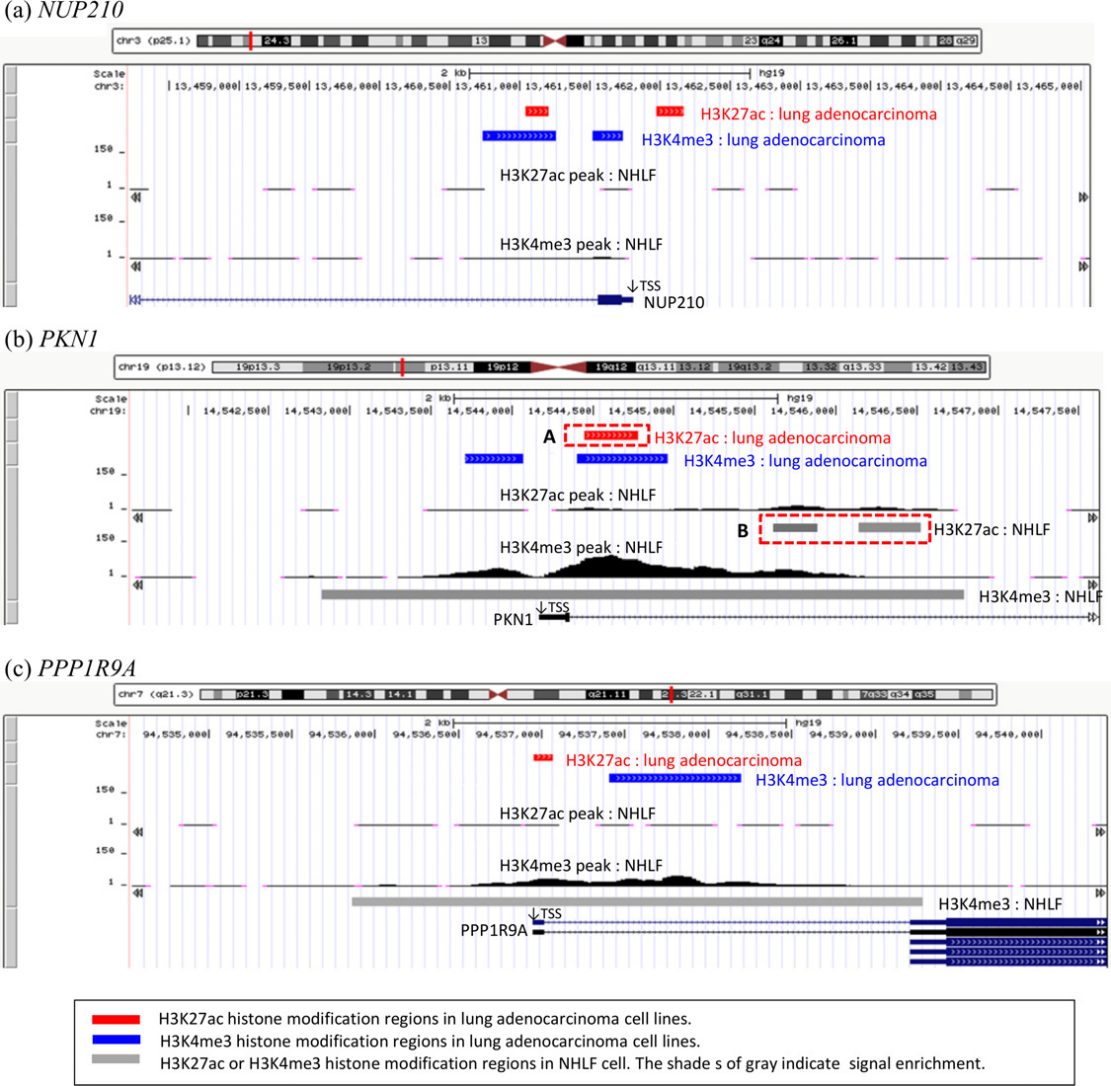
Dual histone modification with high lung adenocarcinoma specificity in another normal cell line. Locations of H3K27ac and H3K4me3 peaks in the normal cell line, NHLF, are shown for the six genes in Table 3: (a) *NUP210* (top), (b) *PKN1*, (c) *PPP1R9A*, (d) *IGF2BP3*, (e) *HOXC4*, and (f) *FAM102B* (bottom). The dashed red rectangles A and B in (c) indicate a difference in location of H3K27ac histone modification between lung adenocarcinoma (chr19:14544446–14544776 on hg19) and NHLF (chr19: 14545611–14545886 and chr19:14546141–14546522 on hg19). Red rectangles indicate H3K27ac histone modifications and blue rectangles indicate H3K4me3 histone modifications that are highly specific to the lung adenocarcinoma cell lines. Gray rectangles are H3K27ac (upper) and H3K4me3 (lower) modifications in NHLF. The shade of gray indicates signal enrichment, which is calculated as the number of sequenced tags overlapping a 25 bp window centered at that position according to UCSC (the darker gray indicates higher numbers). Bottom lines in the figure indicate the location of each gene. H3K27ac and H3K4me3 read coverage in NHLF is shown in a range of 0–150. The genomic positions in this figure are based on hg19.

However, among the six genes in Table 3, *NUP210* did not carry H3K27ac or H3K4me3 marks in its promoter region in NHLF (Fig 2(A)). These results support that these histone modifications are highly specific to lung adenocarcinoma. We also determined that although, in NHLF, another gene *PKN1* (member of the protein kinase C superfamily) carried the dual histone modifications in the promoter region, differences were observed in the location of the H3K27ac histone modifications compared to those highly shared among the lung adenocarcinoma cell lines (Fig 2(B)-A (lung adenocarcinoma) compared to B (NHLF)). The H3K27ac histone modification of *PKN1* was located near the TSS in the lung adenocarcinoma cell lines (chr19:14544446–14544776 on hg19) (Fig 2(B)-A), while it was detected about 1 kb downstream of the TSS in NHLF (chr19: 14545611–14545886 and chr19:14546141–14546522 on hg19) (Fig 2(B)-B). In addition, we determined that *PPP1R9A* (encoding neurabin I in skeletal muscle and neural tissues) did not carry H3K27ac histone modification in its promoter region in NHLF (Fig 2(C)). These results on *PKN1* and *PPP1R9A* partially support the findings of the histone modifications highly specific to lung-adenocarcinoma. However, the other three genes in Table 3 carried both H3K27ac and H3K4me3 marks in their promoter regions in NHLF (S5A, S5B and S5C Fig.

### Gene Function

By focusing on the three genes above (*NUP210*, *PKN1*, and *PPP1R9A*), we examined their functions and known relationships with cancer (Table 3). We also described the results obtained with the other genes in S1–S4 Appendices. *NUP210* encodes a main component protein of the nuclear pore complex, forming a gateway that regulates the flow of macromolecules between the nucleus and the cytoplasm [33]. *PKN1* encodes a protein kinase C super family member, regulating cell migration and gene expression [24]. This protein is associated with ovarian cancer [21], colorectal cancer [22], prostate cancer [23, 24], breast cancer [25], and gastric cancer [26]. *PPP1R9A* encodes neurabin, which is involved in the synapse formation and function. *PPP1R9A* protein is a regulatory subunit of protein phosphatase I and controls actin cytoskeleton reorganization [34]. *PPP1R9A* is upregulated both in benign and cancer prostatic tissues [27].

There has been no report regarding the relationship of these three genes with lung adenocarcinoma. Thus, we examined the association between these three genes and lung adenocarcinoma further in the following sections.

### Gene Expression Levels

Both H3K27ac and H3K4me3 are known to be associated with the activation of gene transcription [35–38]. Thus, we investigated the expression levels (in RPKM) of the three genes (*NUP210*, *PKN1*, and *PPP1R9A*) in each of the lung adenocarcinoma cell lines and compared it with that in SAEC (S6 Table). We used the following criteria [15] of aberrant gene expression: (i) ≥4- or ≤1/16-fold RPKM of SAEC if the genes were transcribed (>1 RPKM) in SAEC and (ii) >5 RPKM if the genes were not transcribed (≤1 RPKM) in SAEC.

**Fig 3:**
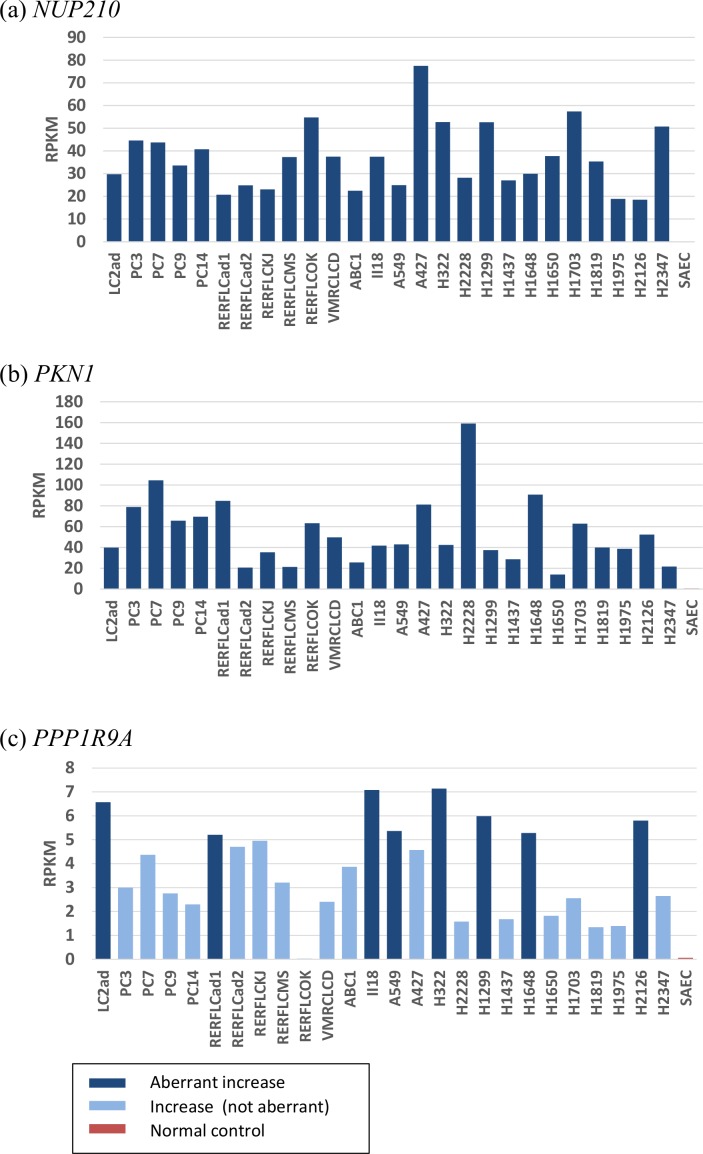
Higher levels of gene expression of the genes with dual histone modifications. RPKM values of three genes in Table 3 are shown for the 26 lung adenocarcinoma cell lines: (a) *NUP210* (top), (b) *PKN1* (middle), and (c) *PPP1R9A* (bottom). Expression levels of the other three genes in Table 3 (*IGF2BP3*, *HOXC4*, and *FAM102B*) are shown in S8 Fig. Navy bars indicate transcriptional aberrations when compared with SAEC. Red bar at the right end shows the expression levels in SAEC. RPKM values at the bottom in the table indicate averages among the 26 lung adenocarcinoma cell lines.

RPKM values of the three genes in each of the 26 lung adenocarcinoma cell lines and SAEC are shown in Fig 3 (for (a) *NUP210*, (b) *PKN1*, and (c) *PPP1R9A*). The number of lung adenocarcinoma cell lines aberrantly expressing *NUP210*, *PKN1*, and *PPP1R9A* was 26, 26, and 8, respectively (Fig 3 and S7 Table).

It is worth noting that *NUP210* and *PKN1* genes were aberrantly expressed in all of the 26 lung adenocarcinoma cells. The average RPKM values of *NUP210*, *PKN1*, and *PPP1R9A* are 37.0, 54.4, and 3.8 (i.e., 75.5, 284.7, and 62.6-fold increase when compared to that of SAEC), respectively.

### Chromatin States of Genes with the Chromatin Marks

We also assessed chromatin states in *NUP210*, *PKN1*, and *PPP1R9A* promoter regions (±1.5 kb from TSS) that were inferred along each genome in the previous study [11]. For each of the three genes, chromatin states most frequently inferred in its promoter region in each cell line are shown in S6 Fig as in [15].

Promoter regions of the *NUP210*, *PKN1*, and *PPP1R9A* genes were estimated to be inactive in SAEC. In contrast, chromatin states of the promoter regions of the three genes were estimated to be “active promoter” in equal to or more than 85% of the 26 lung adenocarcinoma cell lines. These results support that the dual chromatin marks (H3K27ac and H3K4me3) highly specific to lung adenocarcinoma change the chromatin structure, which can lead to the aberrant expression of these genes.

### Examination of Single Nucleotide Polymorphisms in the Promoter and Gene Bodies

Association between gene expression and genetic polymorphisms has been of interest [39]. In order to explore the potential association between the aberrant gene expression described above and genetic polymorphisms, we examined the location of SNPs within the promoter regions (±1.5 kb from TSS) and gene bodies (exons and introns) of *NUP210* and *PKN1*, which were aberrantly expressed in all of the 26 lung adenocarcinoma cell lines. The locations in each of the 26 lung adenocarcinoma cell lines are shown in S7 Fig. However, no SNP was shared in all 26 lung adenocarcinoma cells that could explain the aberrant gene expression of *NUP210* and *PKN1*. These results support that histone modifications are the primary cause of aberrant gene expression in lung adenocarcinoma.

### Comparison of Expression Levels of Histone Modifying Enzymes

In order to explore potential causes of histone modifications highly specific to lung adenocarcinoma, we investigated the expression levels of “writers” and “erasers” of these modifications; histone lysine methyltransferases, histone lysine demethylases [40, 41], histone acetyltransferases, and histone deacetylases [42, 43]. It is conceivable that the dual modifications are caused either by overexpression of the histone lysine methyltransferases and histone acetyltransferases or by underexpression of histone lysine demethylases and histone deacetylases. We analyzed the expression levels of 17, 7, 8, and 11 of histone lysine methyltransferases, histone acetyltransferases, histone lysine demethylases, and histone deacetylases, respectively (S8 Table).

No considerable increase in the gene expression of histone lysine methyltransferases and histone acetyltransferases was detected (according to a criterion ≥2-fold RPKM of SAEC) among more than 22 lung adenocarcinoma cell lines. At most, the gene expression of a histone lysine methyltransferase, *SMYD3*, and a histone acetyltransferase, *HAT1*, was considerably increased among 21 and 11 lung adenocarcinoma cell lines, respectively. Similarly, no considerable decrease in the gene expression of histone lysine demethylase and histone deacetylase was observed (according to a criterion ≤1/4-fold RPKM of SAEC) in more than 22 cell lines. At most, the gene expression of a histone demethylase, *KDM5D*, and a histone deacetylase, *HDAC11*, was considerably decreased among 18 and 4 lung adenocarcinoma cell lines, respectively.

Regarding histone lysine methyltransferases, if we examine a combination of two enzymes *SMYD3* and *MLL2* (Top two lines in S8 Table), the expression of either of them was considerably increased among almost all cell lines, except for RERFLCad2, which is at the left of the table. The RERFLCad2 cell line lacks the H3K4me3 histone modification in all three genes (*NUP210*, *PKN1*, and *PPP1R9A*) (“presence/absence of H3K4me3 modification” in S8 Table). Therefore, the combination of *SMYD3* and *MLL2* could explain the high specificity of H3K4me3 histone modification to lung adenocarcinoma. *SMYD3* has been reported to be highly overexpressed in colon cancer, liver cancer, breast cancer [44, 45], and prostate cancer [46]. Moreover, the elevated expression level of *MLL2* in the breast and colon cells is associated with malignancy in these tissues [47].

In contrast, the high specificity of the H3K27ac histone modification to lung adenocarcinoma could not be similarly explained. We did not find a combination of histone acetyltransferases or histone deacetylases that were overexpressed or underexpressed among the lung adenocarcinoma cell lines, even if we excluded a cell line, A427, that lacks the H3K27ac histone modification among all three genes (*NUP210*, *PKN1*, and *PPP1R9A*) (“presence/absence of H3K27ac modification” in S8 Table).

### Expression Profiles of the Three Genes in Other Types of Cancer and Carcinomas

In the analysis described above, we identified three marker genes, *NUP210*, *PKN1*, and *PPP1R9A*, carrying histone modifications in their promoter regions and showing aberrant gene expression, both of which are highly specific to lung adenocarcinoma. In order to validate the candidate genes, from a viewpoint of gene expression, we also investigated whether the aberrant gene expression of these genes could be observed in other samples of lung adenocarcinoma. We did not conduct similar validation from a viewpoint of histone modification, because there was no available dataset. Concomitantly, to determine whether these markers are specific to lung adenocarcinoma, we investigated whether these genes were aberrantly expressed in other types of cancer and carcinomas.

**Fig 4:**
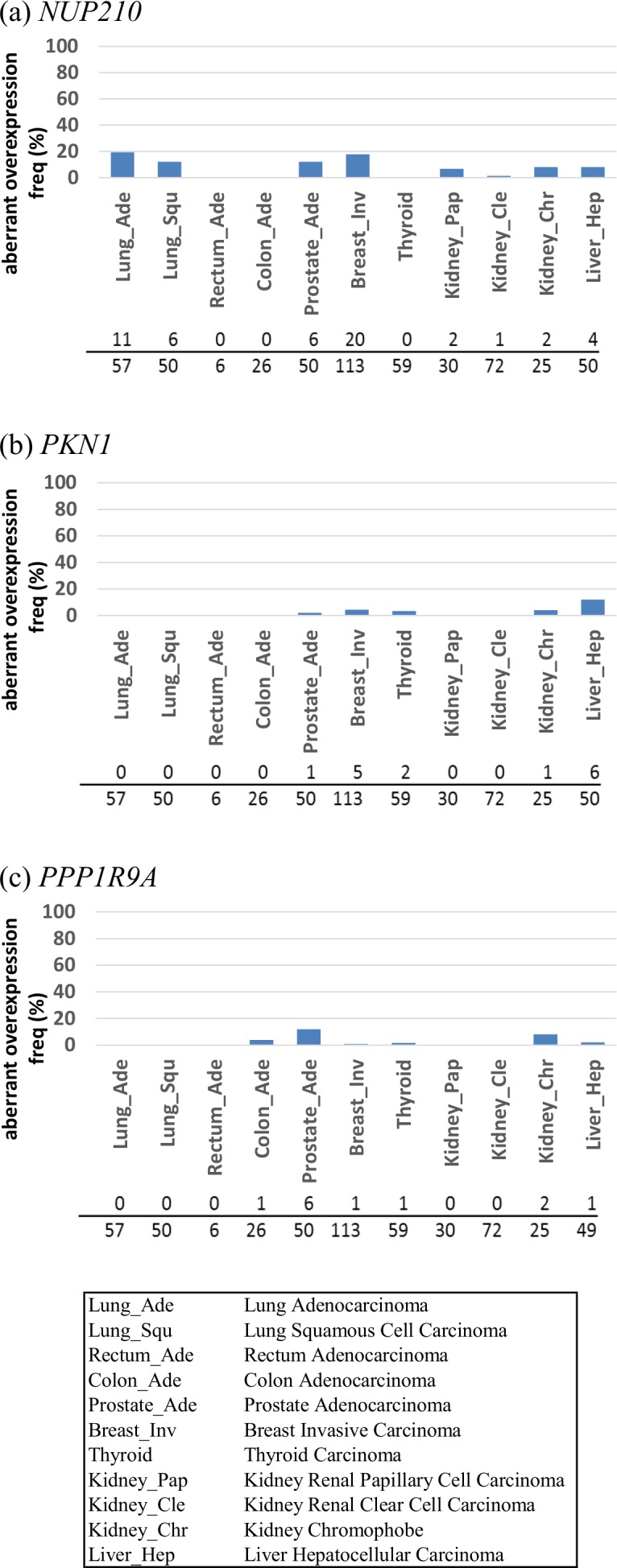
Frequency of aberrant gene expression of the adenocarcinoma-associated genes in several cancer tissue samples. Frequency of aberrant gene expression in several carcinomas obtained from the BioXpress database for three genes in Table 3 are shown: (a) *NUP210* (top), (b) *PKN1* (middle), and (c) *PPP1R9A* (bottom). Similar illustrations of the other three genes in Table 3 (*IGF2BP3*, *HOXC4*, and *FAM102B*) are shown in S7 Fig. These are gene expression data of 538 paired tumors compared with normal tissue samples from the same patients and baseline expression data in normal human tissues. At the bottom of each figure, upper numbers indicate the number of paired tissue samples presenting aberrant expression; lower numbers indicate the total number of paired tissue samples for each type of cancer.

We analyzed RNA-Seq gene expression data of 538 paired tumor and normal tissue samples, which are available in the BioXpress database [17] for 11 different types of cancer, including two types of lung cancer (lung adenocarcinoma [Lung_Ade] and lung squamous cell carcinoma [Lung_Squ]) (Fig 4 and S2 Table). We examined the 11 types of cancer because we could obtain baseline expression data in the corresponding tissue to these cancers (S2 Table).

Similarly to the analysis described above, we used the following criteria [15] of aberrant gene expression: (i) ≥4- or ≤1/16-fold FPKM of baseline expression from each normal tissue if the genes were transcribed (>1 FPKM) in normal tissues and (ii) >5 FPKM if the genes were not transcribed (≤1 FPKM) in normal tissues. We used data of FPKM in each normal tissue as baseline expression data in Expression Atlas baseline database. For example, the FPKM value of *NUP210* in each of the lung adenocarcinoma tissue sample and the normal lung tissue sample is shown in S9 Table, together with the levels in RPKM seen in the cell line data.

**Fig 5:**
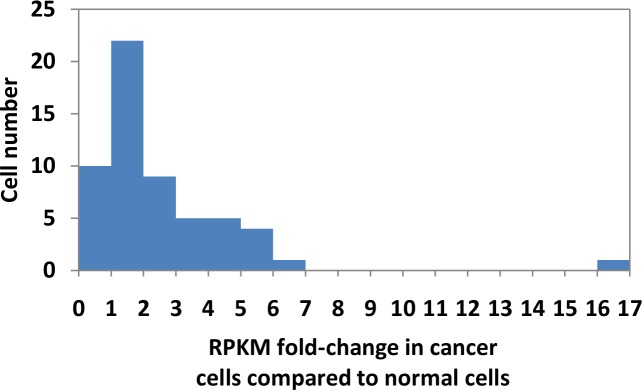
*NUP210* gene expression fold change in lung adenocarcinoma tissue samples. The y-axis indicates the number of paired-samples and the x-axis indicates the gene expression (RPKM) fold-change in cancer versus the paired normal tissue samples. Frequency of fold-change >1, >2, and >3 is about 84%, 44%, and 28% in lung adenocarcinoma tissue samples, respectively.

Frequency of lung adenocarcinoma tissue samples showing aberrant *NUP210* expression was about 19.3% and that of the other 10 types of carcinoma was 0–17.7% (Fig 4(A) and S10 Table). Further examination of its fold-change revealed that the frequency of 57 lung adenocarcinoma tissue samples showing ≥3-, ≥2-, and ≥1-fold RPKM compared to the paired control was about 28%, 44%, and 84%, respectively (Fig 5). These results show that the extent of *NUP210* overexpression in lung adenocarcinoma is usually smaller than that in the 26 lung adenocarcinoma cell lines. Regarding the other two genes (*PKN1* and *PPP1R9A*), although the frequency of aberrant gene expression in the other 10 types of carcinoma was 0–12.0% (*PKN1*) and 0–12.0% (*PPP1R9A*), no lung adenocarcinoma tissue sample showed aberrant gene expression (Fig 4(B) and 4(C) and S10 Table).

## Discussion

The dataset of the 26 lung adenocarcinoma cell lines and the normal cell line, SAEC, is so far the largest published multi-omics dataset. By utilizing it, we determined that the dual modification of H3K27ac and H3K4me3 in the promoter regions was associated with lung adenocarcinoma. These epigenetic modifications are not present in SAEC, but present in ≥85% (22 out of 26) and ≤ 96% (25 out of 26) of the lung adenocarcinoma cell lines. They are more highly specific to cancer cells than the previously reported ones [5] that are still present at low levels in normal cells (11.8±7.1%, 4–12 genes (N = 3), compared to 24.7±4.1%, 49–248 genes (N = 6) in cancer cells).

In previous studies, the sensitivity of DNA methylation biomarkers for gastric cancer was reported to range from 56% to 96% [48] and from 62% to 75% for colorectal cancer or adenomas according to two meta-analyses [49, 50]. In a previous study, human hepatocellular carcinoma (HCC) was predicted with 95.6% sensitivity by quantifying the DNA methylation level in preneoplastic liver tissues [51]. Compared to these studies that explored DNA methylation biomarkers, the sensitivity of epigenetic biomarkers (histone modification) described in the present study is at most 96% (25 out of 26), which is comparable to that reported in the literature.

The dataset was generated and analyzed in a previous study [15]. Compared to our study, the focus was the diversity of mutations and aberrations in the epigenome and transcriptome among the 26 lung adenocarcinoma cell lines. For example, the expression patterns of genes were compared among 26 lung adenocarcinoma cell lines and certain genes aberrantly expressed in at least one lung adenocarcinoma cell line were detected. In contrast, we explored epigenetic modifications highly shared among the 26 lung adenocarcinoma cell lines. In order to validate the results obtained from the dataset, we also analyzed ChIP-Seq data of another normal cell line, NHLF, and RNA-Seq data of 57 lung adenocarcinoma tissue samples.

A limitation of the present study is that we were able to use the data of only two normal cell lines (SAEC and NHLF). No other ChIP-Seq data (including input control) of a normal cell line from an adult lung tissue is available at the International Human Epigenome Consortium (IHEC) database (http://ihec-epigenomes.org/). In addition, we did not analyze DNA methylation because there was no available data of BS-Seq of the two normal cell lines Thus, we did not investigate the association between a certain histone modification and DNA methylation as previously described in a previous study using microarray data [5].

Despite these limitations, to our knowledge, this is the first study revealing epigenetic markers highly specific to lung adenocarcinoma based on multi-omics data at the population level of cancer and normal cells (i.e., dozens of cancer cell lines and multiple normal cell lines). The most important finding was the dual modifications of H3K27ac and H3K4me3 in the promoter region of *NUP210*. The dual modification was associated with its aberrant overexpression in all 26 lung adenocarcinoma cell lines, although the validation analysis showed that the extent of overexpression was smaller in other lung adenocarcinoma tissue samples There is a statistically significant difference in terms of age and sex between the lung adenocarcinoma tissue samples and the cell lines (S1 and S2 Tables) (P = 0.0002 by Wilcoxon rank sum test for age, P = 0.02 by Chi-square test for sex), which could be a factor behind it. *NUP210* mediates muscle cell differentiation by regulating nuclear envelope (NE) endoplasmic reticulum homeostasis [52] and a recent study of NE proteins in cancer samples revealed that *NUP210* is usually upregulated in tumors (particularly in ovary, breast, and prostate) [53]. In order to increase its reliability as a biomarker, further studies are warranted to systematically investigate the presence or absence of the dual histone modification in the promoter of *NUP210* among other lung adenocarcinoma cells and normal cells.

The present study provides a basis to discover epigenetic biomarkers highly specific to a specific type of cancer for diagnosis and epigenetic therapy based on the multi-omics data at the cell population level.

## Supporting Information

S1 Appendix Function of the genes in Tables 2 and 3 not described in the main manuscript. (DOCX)

S2 Appendix Expression levels of the genes with chromatin marks listed in Tables 2 and 3, but not discussed in the text. (DOCX)

S3 Appendix Chromatin states of the genes with the chromatin marks listed in Tables 2 and 3, but not discussed in the text. (DOCX)

S4 Appendix Expression levels for the genes listed Table 2 and 3, but not discussed in the text, in other types of cancer. (DOCX)

S1 Fig The number of histone-modifications not detected in SAEC, but observed in each of the 26 lung adenocarcinoma cell lines. The number of six types of histone modifications not detected in SAEC, but observed in each of the 26 lung adenocarcinoma cell lines is shown. (a) H3K27ac (top left), (b) H3K4me3 (top right), (c) H3K4me1 (middle left), (d) H3K9me3 (middle right), (e) H3K36me3 (bottom left), and (f) H3K27me3 (bottom right).
(TIFF)

S2 Fig Read coverage of H3K4me3 histone modifications of the four genes shared among all lung adenocarcinoma cell lines. H3K4me3 histone modifications are located in the promoter regions of (a) *NFE2L3*, (b) *ETV4*, (c) *PRTG*, and (d) *TMEM86A*. The blue arrows indicate the locations of the four genes and the red arrows indicate regions of the histone modifications. The Small Airway Epithelial Cell (SAEC) at the bottom is a normal control cell line and the others are the 26 lung adenocarcinoma cell lines. H3K4me3 read coverage is shown in a range of 0–300 in (a), 0–400 in (b), 0–500 in (c), and 0–300 in (d), respectively. The genomic positions in this figure are based on hg38.
(TIFF)

S3 Fig Read coverage of H3K27ac and H3K4me3 histone modifications of the four genes specific to the 26 lung adenocarcinoma cell lines. H3K27ac and H3K4me3 histone modifications specific to the 26 lung adenocarcinoma cell lines are located in the promoter regions of (a) *PPP1R9A*, (b) *IGF2BP3*, (c) *HOXC4*, and (d) *FAM102B*. The left panels represent H3K27ac and the right panels represent H3K4me3 modifications. The blue arrows indicate the locations of the four genes and the red arrows indicate regions of histone modifications. Small Airway Epithelial Cell line (SAEC) at the bottom is a normal control cell line and the others are the 26 lung adenocarcinoma cell lines. H3K27ac and H3K4me3 read coverage is shown in a range of 0–150 and 0–300 in (a), 0–300, and 0–500 in (b), 0–300 and 0–500 in (c), and 0–100 and 0–500 in (d), respectively. The genomic positions in this figure are based on hg38.
(TIFF)

S4 Fig The H3K4me3 histone modifications shared among all lung adenocarcinoma cell lines in another normal cell line. Locations of H3K4me3 peaks in a normal cell line, Normal adult Human Lung Fibroblasts (NHLF) are shown for four genes in Table 2: (a) NFE2L3 (top), (b) ETV4, (c) PRTG, and (d) TMEM86A (bottom). Blue rectangles indicate H3K4me3 histone modifications in lung adenocarcinoma cell lines and gray rectangles are H3K4me3 modifications in NHLF. The shade of gray indicates signal enrichment, which is calculated as the number of sequenced tags overlapping a 25 bp window centered at that position according to UCSC (the darker gray indicates higher numbers). Bottom lines in the figure indicate location of each gene. All H3K4me3 read coverage in NHLF is shown in a range of 0–150. The genomic positions in this figure are based on hg19.
(TIFF)

S5 Fig H3K27ac and H3K4me3 histone modifications highly specific to lung adenocarcinoma cell lines but present in another normal cell line. Locations of H3K27ac and H3K4me3 peaks in a normal cell line, Normal adult Human Lung Fibroblasts (NHLF) are shown for the three genes in Table 3: (a) *IGF2BP3* (top), (b) *HOXC4* (middle), and (c) *FAM102B* (bottom). Red rectangles indicate H3K27ac histone modifications and blue rectangles indicate H3K4me3 histone modifications that are highly specific to the lung adenocarcinoma cell lines. Gray rectangles are H3K27ac (upper) and H3K4me3 (lower) modifications in NHLF. The shade of gray indicates signal enrichment, which is calculated as the number of sequenced tags overlapping a 25 bp window centered at that position according to UCSC (the darker gray indicates higher numbers). Bottom lines in the figure indicate location of each gene. All H3K27ac and H3K4me3 read coverage in NHLF is shown in a range of 0–150. The genomic positions in this figure are based on hg19.
(TIFF)

S6 Fig Chromatin state maps of the promoter regions from ten genes. Chromatin states inferred by ChromHMM for the genes listed in Table 2 (top) and Table 3 (bottom) are shown. Chromatin states that most frequently appeared in the promoter were drawn for each gene and cell line. Two chromatin states were drawn when they appeared with equivalent frequency. The color meaning is provided below the maps.
(TIFF)

S7 Fig The distribution of SNPs in the promoters and in the gene bodies of two genes. Distributions of SNPs in each of the 26 lung adenocarcinoma cell lines are shown for two genes listed in Table 3: (a) *NUP210* (top), (b) *PKN1* (bottom). The left panels represent the promoter regions and the right panels represent the gene bodies (exons and introns). The bar lines indicate the SNP locations. The blue arrows indicate the locations of each gene.
(TIFF)

S8 Fig High levels of expression of the genes with H3K4me3 histone modifications in all 26 lung adenocarcinoma cell lines and with dual histone modifications highly specific to lung adenocarcinoma cell lines. The RPKM values of four genes in Table 2 and three genes in Table 3 are shown for the 26 lung adenocarcinoma cell lines: (a) *NFE2L3*, (b) *ETV4*, (c) *PRTG*, (d) *TMEM86A*, (e) *IGF2BP3*, (f) *HOXC4*, and (g) *FAM102B*. Navy bars indicate transcriptional aberrations compared with SAEC. The red bar at the right end shows expression levels in SAEC. RPKM values at the bottom in the table indicate averages among the 26 lung adenocarcinoma cell lines.
(TIFF)

S9 Fig Frequency of aberrant gene expression of the adenocarcinoma-associated genes in other cancer tissue samples. The expression levels of three genes listed in Table 2 and three genes listed in Table 3 are shown: (a) *NFE2L3* (top left), (b) *PRTG* (top right), (c) *TMEM86A* (middle left), (d) *PPP1R9A* (middle right), (e) *HOXC4* (bottom left), and (f) *FAM102B* (bottom right).
(TIFF)

S1 Table Twenty six lung adenocarcinoma cell lines and a human normal small airway epithelial cell line (SAEC). (XLSX)

S2 Table List of tissue samples for examination of gene expression levels in other types of cancer. (XLSX)

S3 Table Number of histone-modifications not detected in SAEC, but observed in each of the 26 lung adenocarcinoma cell lines. (XLSX)

S4 Table List of genes with the H3K4me3 mark in their promoter region of equal to or more than 85% (22 out of 26) of the cancer cell lines. (XLSX)

S5 Table List of genes with the H3K27ac mark in their promoter in equal to or more than 85% (22 out of 26) of the cancer cell lines. (XLSX)

S6 Table Gene expression levels (RPKM) and their average among the 26 adenocarcinoma cell lines. (XLSX)

S7 Table Frequency of aberrant gene expression among the 26 adenocarcinoma cell lines. (XLSX)

S8 Table List of histone modifying enzymes and fold-change of their expression levels when compared to that in SAEC. (XLSX)

S9 Table Expression levels of *NUP210* in the tissue samples and the cell lines. (XLSX)

S10 Table Frequency of aberrant gene expression of the lung adenocarcinoma-associated genes in other cancer tissue samples. (XLSX)
