# Supplementary material for: Identification of Epigenetic Biomarkers of Lung Adenocarcinoma through Multi-Omics Data Analysis
Source: PLoS One. 2016 Apr 4;11(4):e0152918. doi: 10.1371/journal.pone.0152918 (PMC4820141; doi:10.1371/journal.pone.0152918)
Supplement: S1 Appendix — (DOCX) [file pone.0152918.s001.docx]

**S1 Appendix. Function of the genes in Table 2 and 3 not described in the main manuscript.**

For four genes in Table 2 and three genes (*IGF2BP3*, *HOXC4,* and *FAM102B*) in Table3, which were not described in the text, we examined their functions and known relationships with various cancers (Table 2 and Table 3).

*NFE2L3* encodes a member of the cap ‘n’ collar protein family associated with various cellular processes, including differentiation, stress response, inflammation, and carcinogenesis [46, 47]. *NFE2L3* expression is upregulated in colorectal cancer cells [48]. *ETV4* encodes an E-Twenty-Six (ETS) transcription factor of oncogenes and is associated with several cancers, including breast cancer, Ewing tumor, prostate cancer, colorectal cancer, gastric adenocarcinoma, esophageal adenocarcinoma, ovarian cancer, oral squamous cell carcinoma, and non-small-cell lung cancer [49]. *PRTG* encodes protogenin, which belongs to the immunoglobulin superfamily expressed in the developing nervous system [50, 51] and is associated with colorectal cancer [52].

*IGF2BP3* encodes a protein, which can bind to the 5' UTR of the insulin-like growth factor II leader 3 mRNA and plays important roles in cell migration, differentiation, and proliferation [19]. *IGF2BP3* protein is related to about 50 human cancers in 13 organs and is upregulated in lung cancer [53, 54]. Its gene expression is associated with higher tumor grading and is usually elevated in small cell and metastatic lung cancers [53, 54]. *HOXC4* encodes one of the homeobox family members and functions as a transcription factor. Genes from the HOXC cluster, including *HOXC4,* are upregulated in malignant prostate cell lines and lymph node metastases [55].
