# Supplementary material for: Identification of Epigenetic Biomarkers of Lung Adenocarcinoma through Multi-Omics Data Analysis
Source: PLoS One. 2016 Apr 4;11(4):e0152918. doi: 10.1371/journal.pone.0152918 (PMC4820141; doi:10.1371/journal.pone.0152918)
Supplement: S2 Appendix — (DOCX) [file pone.0152918.s002.docx]

**S****2 Appendix. Expression levels of the genes with chromatin marks listed in Table 2 and 3, but not discussed in the text.**

We investigated the expression levels (in RPKM) of seven genes (*NFE2L3*, *ETV4*, *PRTG*, *TMEM86A*, *IGF2BP3*, *HOXC4,* and *FAM102B*) not discussed in the text in each lung adenocarcinoma cell line, in comparison with that in SAEC (S6 Table). We used the following criteria [15] of aberrant gene expression: (i) $\geq$4- or $\leq$1/16-fold RPKM of SAEC if the genes were transcribed (>1 RPKM) in SAEC and (ii) >5 RPKM if the genes were not transcribed ($\leq$1 RPKM) in SAEC.

The number of lung adenocarcinoma cell lines showing the aberrant gene expression was 9 (*NFE2L3*), 19 (*ETV4*), 1 (*PRTG*), 2 (*TMEM86A*) 22 (*IGF2BP3*), 14 (*HOXC4*), and 7 (*FAM102B*), respectively (S7 Table). Of four genes with H3K4me3 histone modifications that were shared among all lung adenocarcinoma cell lines, no gene was aberrantly expressed in all 26 lung adenocarcinoma cell lines. *IGF2BP3* gene was aberrantly expressed in 85% of the lung adenocarcinoma cell lines. RPKM values of the seven genes in each of the 26 lung adenocarcinoma cell lines and SAEC are shown in S8 Figure. In contrast, the other two genes (*HOXC4* and *FAM102B*) were not aberrantly expressed in equal to or more than 85% of the lung adenocarcinoma cell lines. RPKM values of the seven genes in each of the 26 lung adenocarcinoma cell lines and SAEC are shown in S8 Figure.
