# Supplementary material for: Identification of Epigenetic Biomarkers of Lung Adenocarcinoma through Multi-Omics Data Analysis
Source: PLoS One. 2016 Apr 4;11(4):e0152918. doi: 10.1371/journal.pone.0152918 (PMC4820141; doi:10.1371/journal.pone.0152918)
Supplement: S3 Appendix — (DOCX) [file pone.0152918.s003.docx]

**S****3 Appendix. Chromatin states of the genes with the chromatin marks listed in Table 2 and 3, but not discussed in the text.**

For the four genes listed in Table 2 and three genes (*IGF2BP3*, *HOXC4,* and *FAM102B*) with the dual histone modifications listed in Table 3, which were not discussed in the text, we also checked chromatin states in their promoter regions (±1.5 kb from TSS).

Chromatin states of the promoter regions of the three genes with H3K4me3 histone modifications shared among all of the lung adenocarcinoma cell lines (*NFE2L3*, *ETV4,* and *TMEM86A*) were estimated to be not active in SAEC. The chromatin structure of these three genes may not be modified by only H3K4me3 histone modifications in their promoter regions. Chromatin states of the promoter region of *ETV4* were estimated to inactivate the promoter in all 26 lung adenocarcinoma cell lines. On the other hand, chromatin states of the promoter region of *PRTG* were estimated to activate the promoter among 23 lung adenocarcinoma cell lines. These two genes were aberrantly expressed in 19 and 1 lung adenocarcinoma cell lines, respectively. The results of chromatin states could not support the gene expression levels (upper table of S6 Figure).

Promoter regions of the *IGF2BP3* and *HOXC4* genes (i.e., except for *FAM102B* among the three genes with dual modifications) were estimated to be inactive in SAEC. In contrast, chromatin states of the promoter regions of the two genes were estimated to be “active promoter” in equal to or more than 85% of the 26 lung adenocarcinoma cell lines. These results support that the dual chromatin marks (H3K27ac and H3K4me3) highly specific to lung adenocarcinoma change the chromatin structure, which may lead to the aberrant gene expression (lower table of S6 Figure).
