# Supplementary material for: Identification of Epigenetic Biomarkers of Lung Adenocarcinoma through Multi-Omics Data Analysis
Source: PLoS One. 2016 Apr 4;11(4):e0152918. doi: 10.1371/journal.pone.0152918 (PMC4820141; doi:10.1371/journal.pone.0152918)
Supplement: S4 Appendix — (DOCX) [file pone.0152918.s004.docx]

**S****4 Appendix. Expression levels for the genes listed Table 2 and 3, but not discussed in the text, in other types of cancer.**

For three genes (*NFE2L3*, *PRTG,* and *TMEM86A*), listed in Table 2 (We could not obtain baseline expression data for *ETV4*) and three genes (*IGF2BP3*, *HOXC4,* and *FAM102B*) with the dual histone modifications listed in Table 3, we examined the gene expression data of 538 pairs of tumor and normal tissue samples from the same patients, which are available in the BioXpress database [17] for 11 different types of cancer, including two types of lung cancer (lung adenocarcinoma [Lung_Ade] and lung squamous cell carcinoma [Lung_Squ]), in comparison with baseline expression (S9 Figure and S2 Table). We used the following criteria [15] of aberrant gene expression: (i) $\geq$4- or $\leq$1/16-fold FPKM of baseline expression if the genes were transcribed (>1 FPKM) in normal tissue and (ii) >5 FPKM if the genes were not transcribed ($\leq$1 FPKM) in normal tissue.

The frequency of lung adenocarcinoma cell lines aberrantly expressing the seven genes was about 24.6%, 0%, 0%, 47.4%, 3.5%, and 1.8 %. Thus, there was no gene highly specific to lung adenocarcinoma aberrantly expressed. *IGF2BP3* was overexpressed in most lung adenocarcinoma samples. These results partially support the previous findings of the relationship between histone modifications and upregulation of gene expression. *NFE2L3* was aberrantly expressed in rectum adenocarcinoma and colon adenocarcinoma, but not in lung adenocarcinoma. *HOXC4* was aberrantly and specifically expressed in prostate adenocarcinoma, which was consistent with previous reports [55, 56].
