# Supplementary figures and images for: Identification of Epigenetic Biomarkers of Lung Adenocarcinoma through Multi-Omics Data Analysis
Source: PLoS One. 2016 Apr 4;11(4):e0152918. doi: 10.1371/journal.pone.0152918 (PMC4820141; doi:10.1371/journal.pone.0152918)

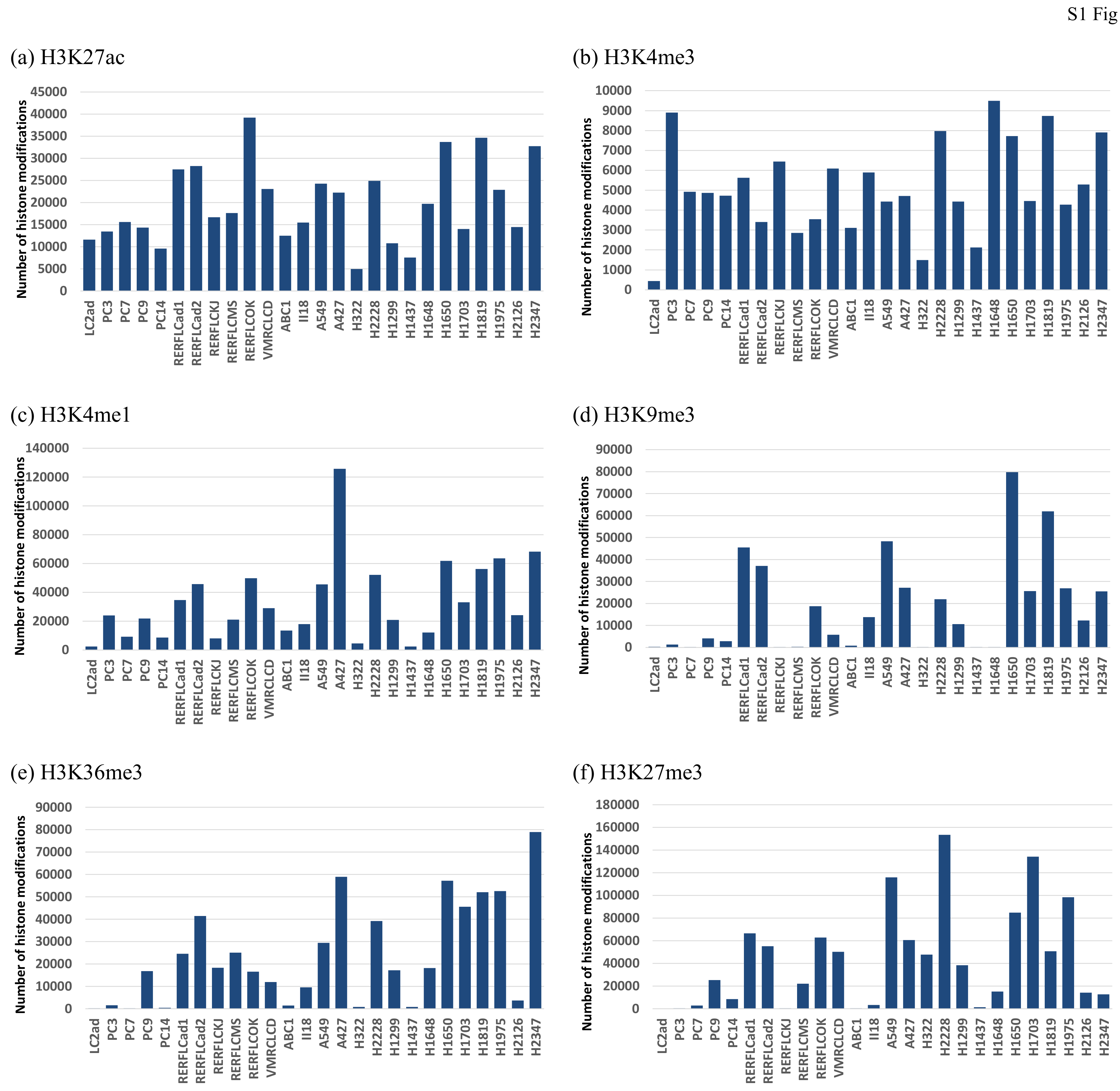

Supplement: S1 Fig — The number of six types of histone modifications not detected in SAEC, but observed in each of the 26 lung adenocarcinoma cell lines is shown. (a) H3K27ac (top left), (b) H3K4me3 (top right), (c) H3K4me1 (middle left), (d) H3K9me3 (middle right), (e) H3K36me3 (bottom left), and (f) H3K27me3 (bottom right). (TIFF) [file pone.0152918.s005.tiff]

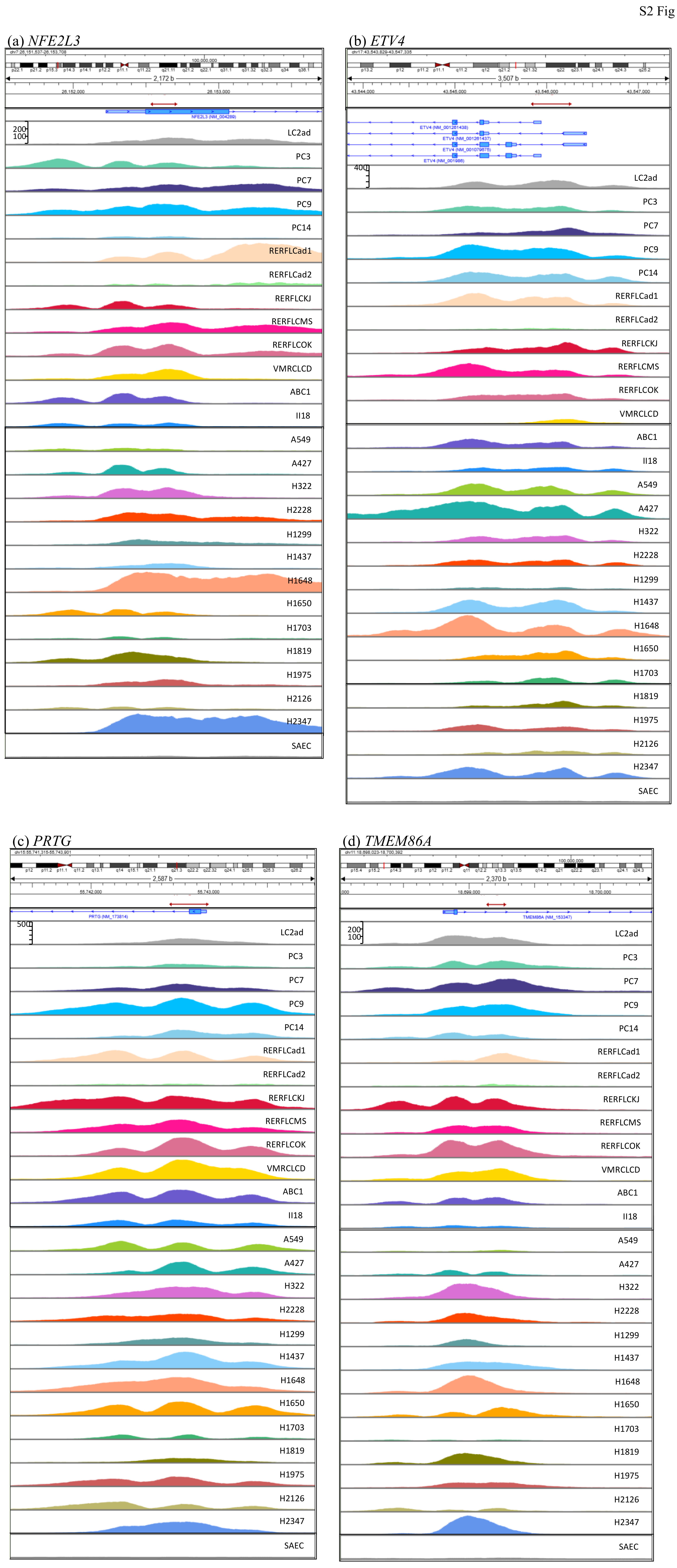

Supplement: S2 Fig — H3K4me3 histone modifications are located in the promoter regions of (a) NFE2L3, (b) ETV4, (c) PRTG, and (d) TMEM86A. The blue arrows indicate the locations of the four genes and the red arrows indicate regions of the histone modifications. The Small Airway Epithelial Cell (SAEC) at the bottom is a normal control cell line and the others are the 26 lung adenocarcinoma cell lines. H3K4me3 read coverage is shown in a range of 0–300 in (a), 0–400 in (b), 0–500 in (c), and 0–300 in (d), respectively. The genomic positions in this figure are based on hg38. (TIFF) [file pone.0152918.s006.tiff]

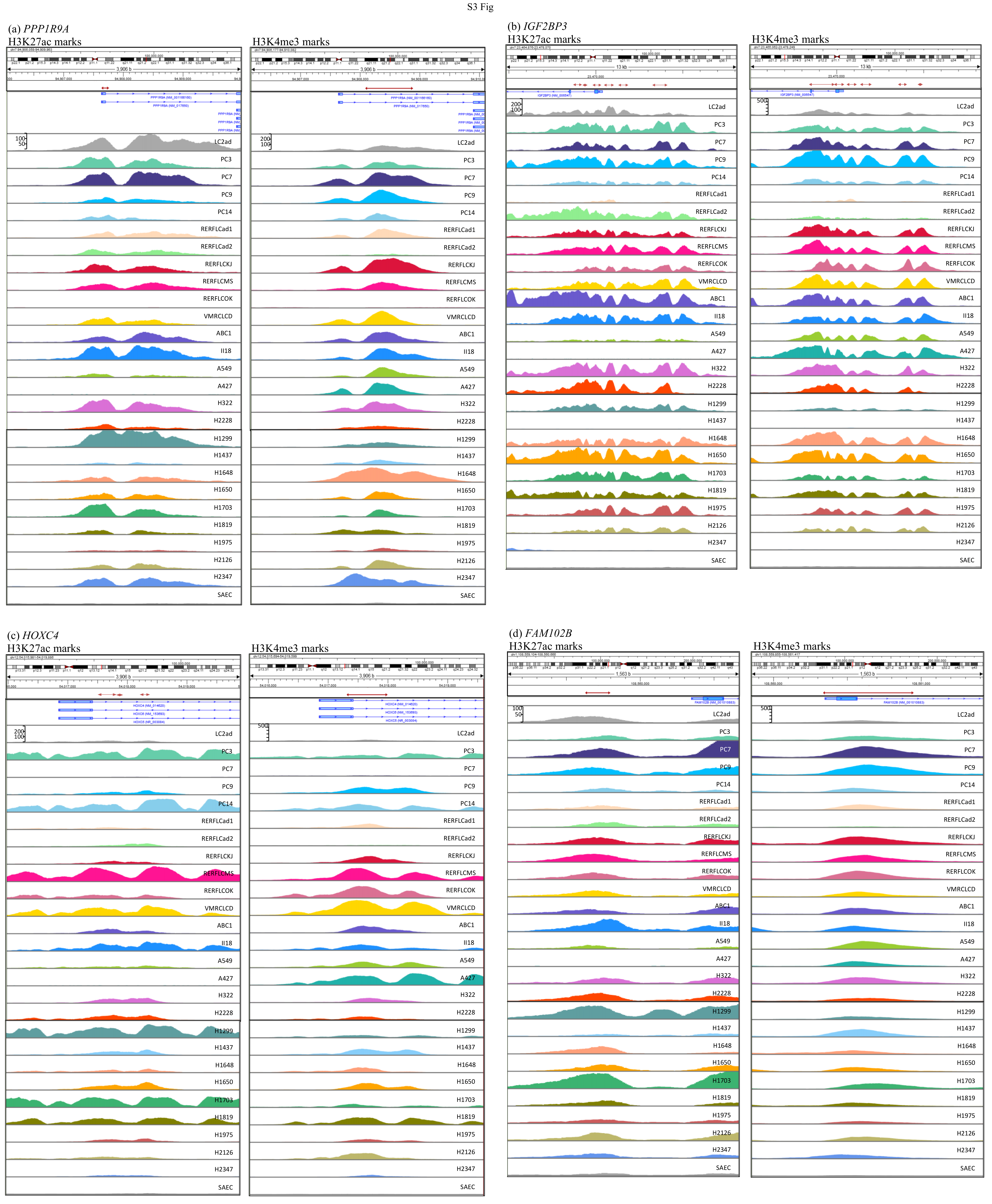

Supplement: S3 Fig — H3K27ac and H3K4me3 histone modifications specific to the 26 lung adenocarcinoma cell lines are located in the promoter regions of (a) PPP1R9A, (b) IGF2BP3, (c) HOXC4, and (d) FAM102B. The left panels represent H3K27ac and the right panels represent H3K4me3 modifications. The blue arrows indicate the locations of the four genes and the red arrows indicate regions of histone modifications. Small Airway Epithelial Cell line (SAEC) at the bottom is a normal control cell line and the others are the 26 lung adenocarcinoma cell lines. H3K27ac and H3K4me3 read coverage is shown in a range of 0–150 and 0–300 in (a), 0–300, and 0–500 in (b), 0–300 and 0–500 in (c), and 0–100 and 0–500 in (d), respectively. The genomic positions in this figure are based on hg38. (TIFF) [file pone.0152918.s007.tiff]

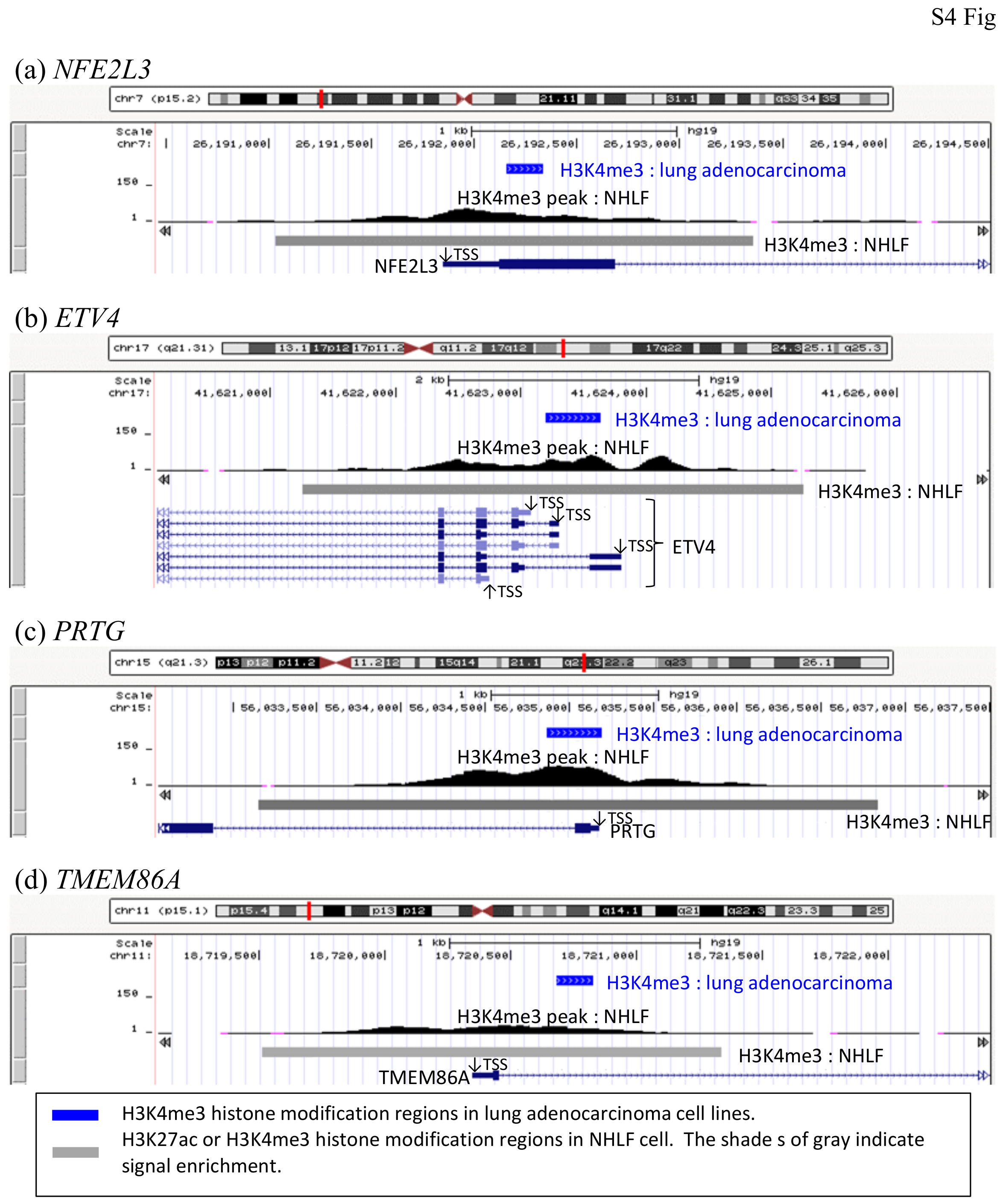

Supplement: S4 Fig — Locations of H3K4me3 peaks in a normal cell line, Normal adult Human Lung Fibroblasts (NHLF) are shown for four genes in Table 2: (a) NFE2L3 (top), (b) ETV4, (c) PRTG, and (d) TMEM86A (bottom). Blue rectangles indicate H3K4me3 histone modifications in lung adenocarcinoma cell lines and gray rectangles are H3K4me3 modifications in NHLF. The shade of gray indicates signal enrichment, which is calculated as the number of sequenced tags overlapping a 25 bp window centered at that position according to UCSC (the darker gray indicates higher numbers). Bottom lines in the figure indicate location of each gene. All H3K4me3 read coverage in NHLF is shown in a range of 0–150. The genomic positions in this figure are based on hg19. (TIFF) [file pone.0152918.s008.tiff]

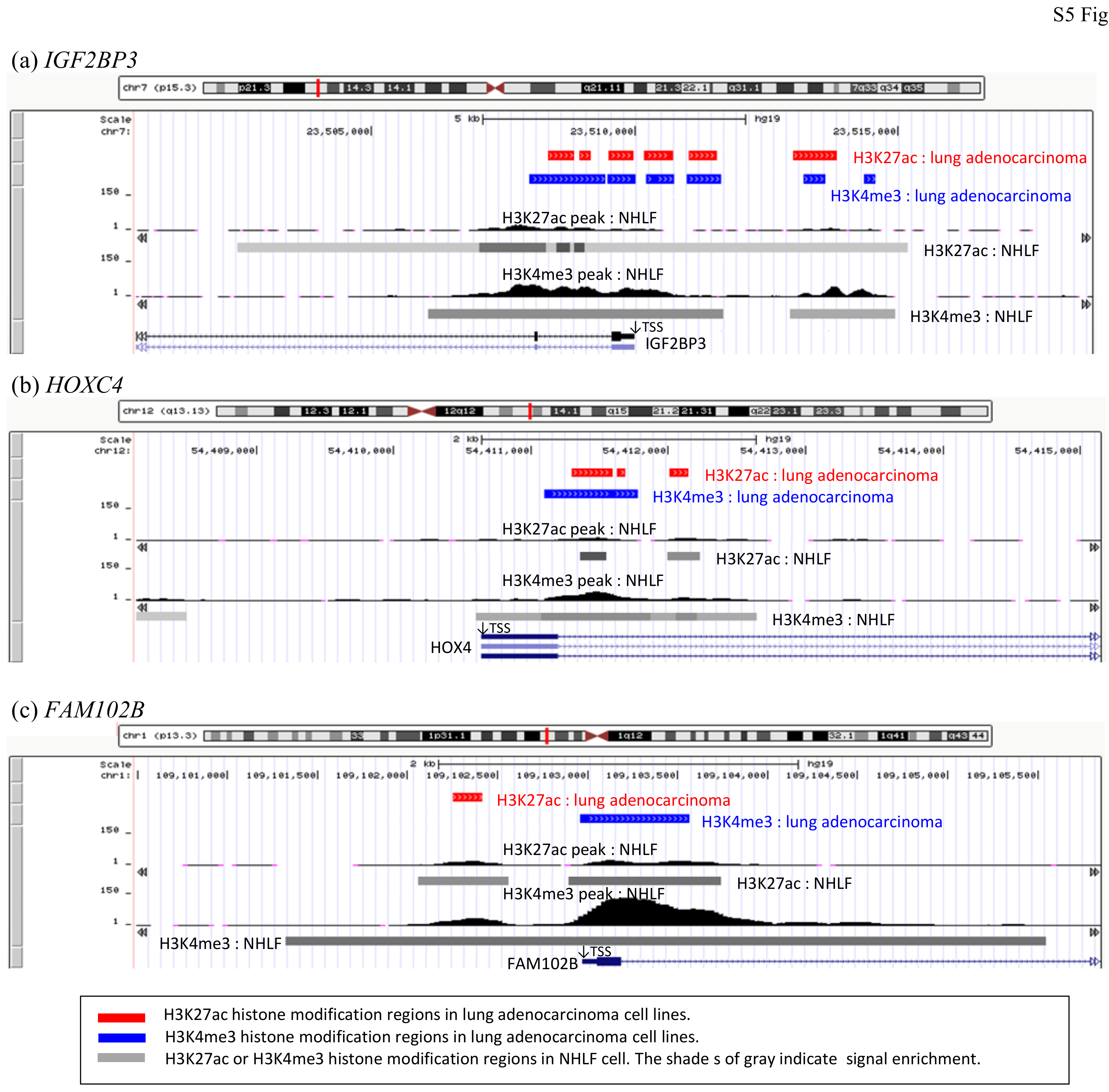

Supplement: S5 Fig — Locations of H3K27ac and H3K4me3 peaks in a normal cell line, Normal adult Human Lung Fibroblasts (NHLF) are shown for the three genes in Table 3: (a) IGF2BP3 (top), (b) HOXC4 (middle), and (c) FAM102B (bottom). Red rectangles indicate H3K27ac histone modifications and blue rectangles indicate H3K4me3 histone modifications that are highly specific to the lung adenocarcinoma cell lines. Gray rectangles are H3K27ac (upper) and H3K4me3 (lower) modifications in NHLF. The shade of gray indicates signal enrichment, which is calculated as the number of sequenced tags overlapping a 25 bp window centered at that position according to UCSC (the darker gray indicates higher numbers). Bottom lines in the figure indicate location of each gene. All H3K27ac and H3K4me3 read coverage in NHLF is shown in a range of 0–150. The genomic positions in this figure are based on hg19. (TIFF) [file pone.0152918.s009.tiff]

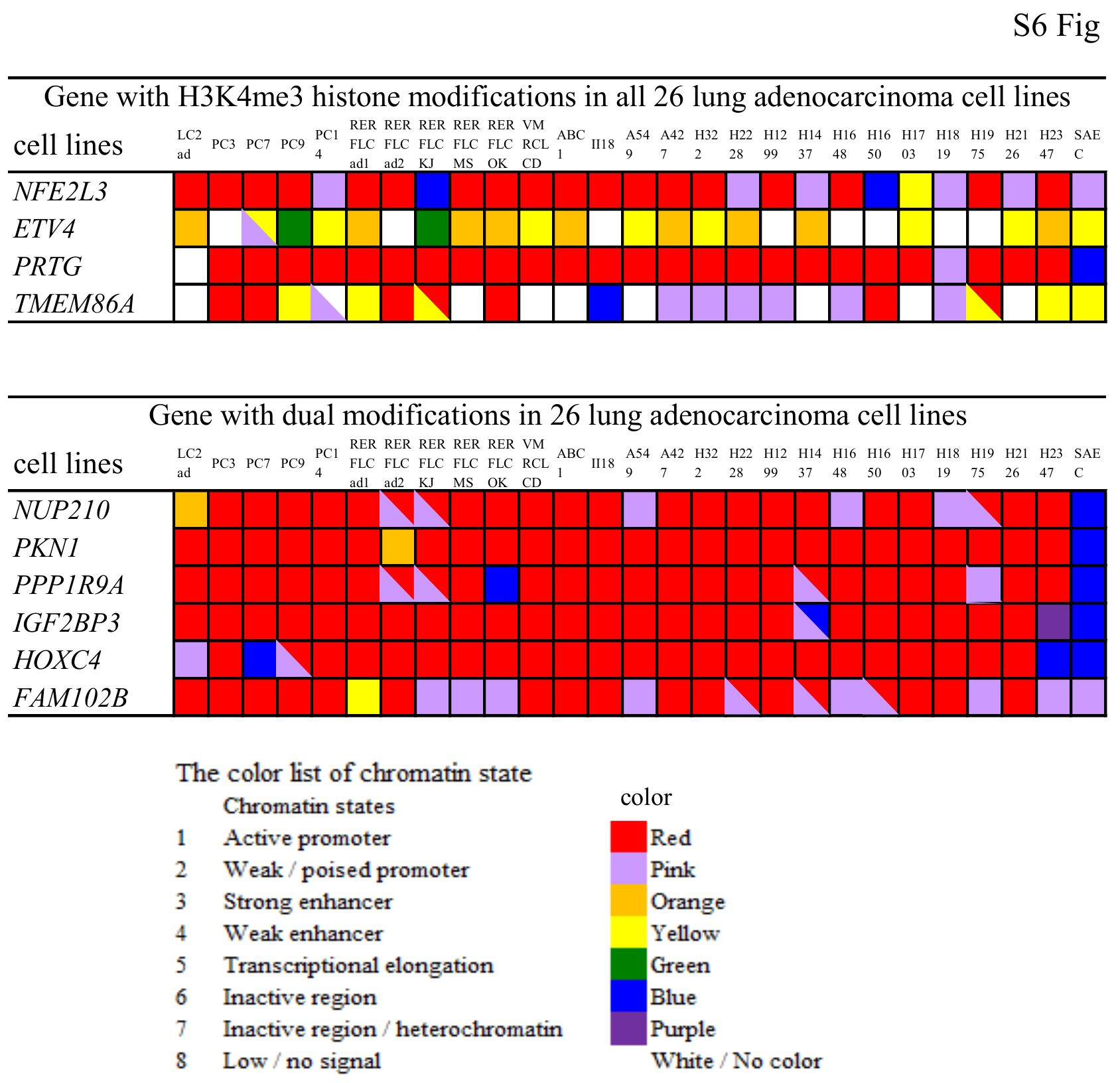

Supplement: S6 Fig — Chromatin states inferred by ChromHMM for the genes listed in Table 2 (top) and Table 3 (bottom) are shown. Chromatin states that most frequently appeared in the promoter were drawn for each gene and cell line. Two chromatin states were drawn when they appeared with equivalent frequency. The color meaning is provided below the maps. (TIFF) [file pone.0152918.s010.tiff]

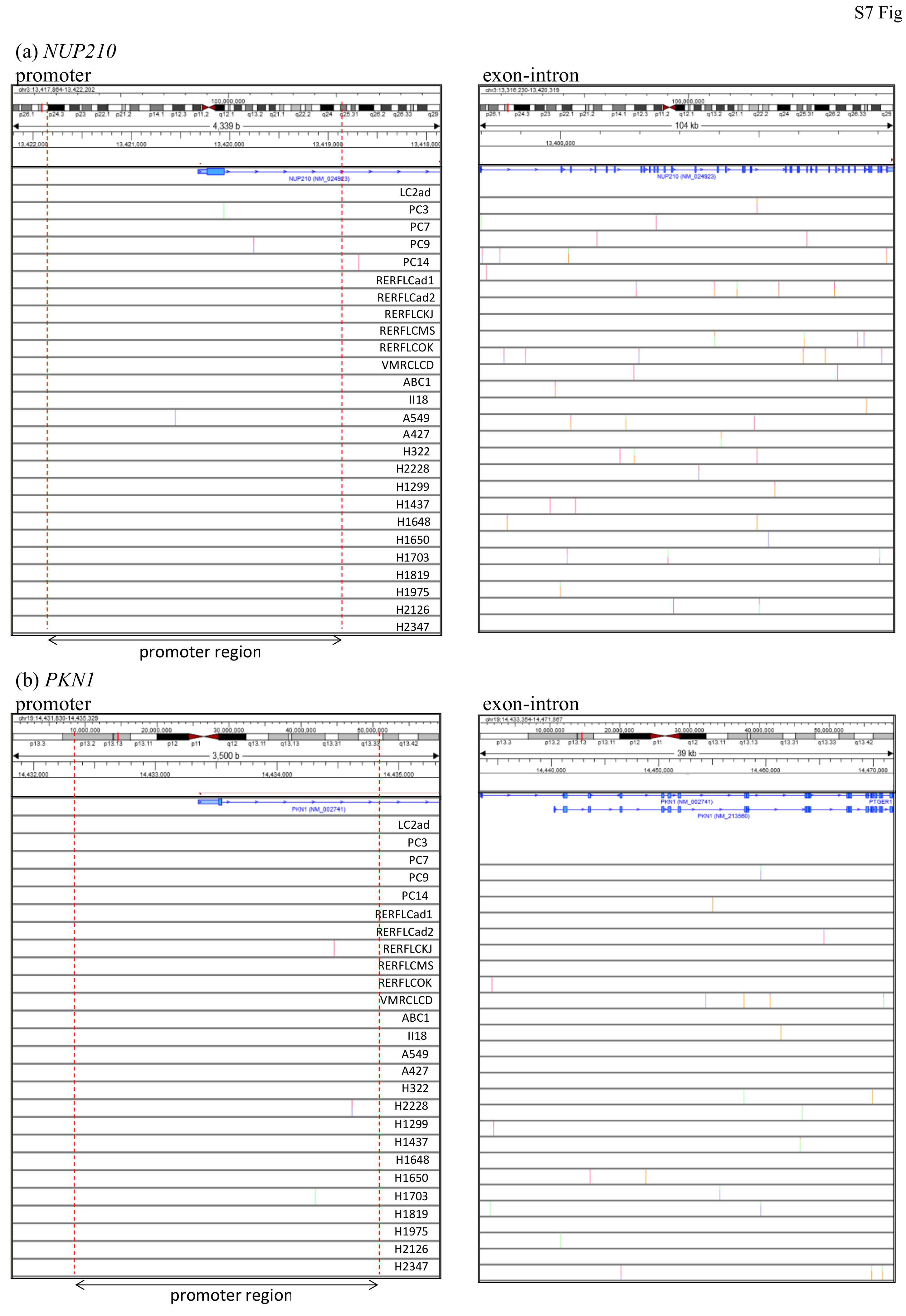

Supplement: S7 Fig — Distributions of SNPs in each of the 26 lung adenocarcinoma cell lines are shown for two genes listed in Table 3: (a) NUP210 (top), (b) PKN1 (bottom). The left panels represent the promoter regions and the right panels represent the gene bodies (exons and introns). The bar lines indicate the SNP locations. The blue arrows indicate the locations of each gene. (TIFF) [file pone.0152918.s011.tiff]

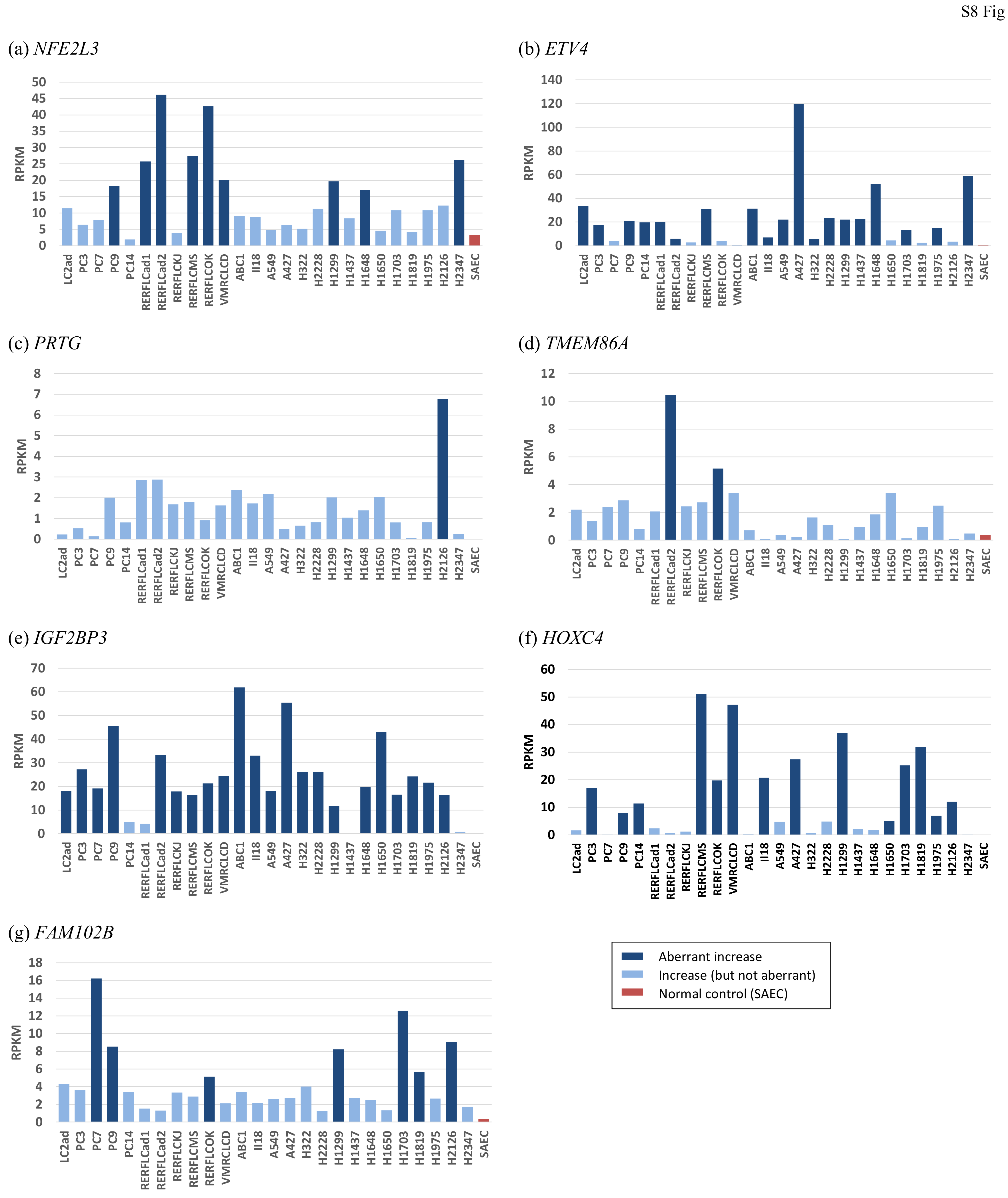

Supplement: S8 Fig — The RPKM values of four genes in Table 2 and three genes in Table 3 are shown for the 26 lung adenocarcinoma cell lines: (a) NFE2L3, (b) ETV4, (c) PRTG, (d) TMEM86A, (e) IGF2BP3, (f) HOXC4, and (g) FAM102B. Navy bars indicate transcriptional aberrations compared with SAEC. The red bar at the right end shows expression levels in SAEC. RPKM values at the bottom in the table indicate averages among the 26 lung adenocarcinoma cell lines. (TIFF) [file pone.0152918.s012.tiff]

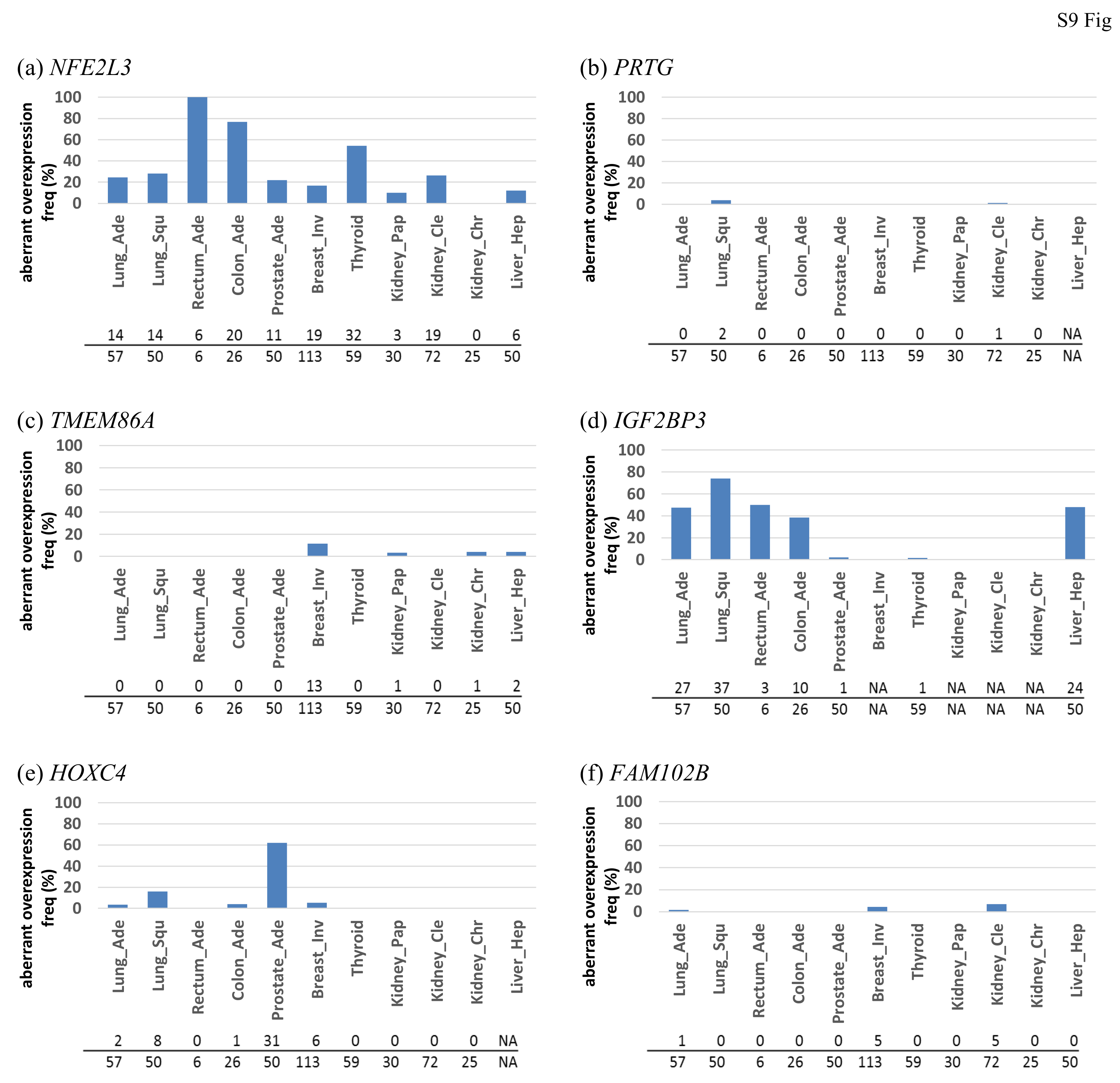

Supplement: S9 Fig — The expression levels of three genes listed in Table 2 and three genes listed in Table 3 are shown: (a) NFE2L3 (top left), (b) PRTG (top right), (c) TMEM86A (middle left), (d) PPP1R9A (middle right), (e) HOXC4 (bottom left), and (f) FAM102B (bottom right). (TIFF) [file pone.0152918.s013.tiff]
